# Brain and Language: Evidence for Neural Multifunctionality

**DOI:** 10.1155/2014/260381

**Published:** 2014-06-09

**Authors:** Dalia Cahana-Amitay, Martin L. Albert

**Affiliations:** Boston University Medical School Department of Neurology, Harold Goodglass Aphasia Research Center & Language in the Aging Brain, Veterans Affairs Boston Healthcare System, 150 South Huntington Avenue (12A), Boston, MA 02130, USA

## Abstract

This review paper presents converging evidence from studies of brain damage and longitudinal studies of language in aging which supports the following thesis: the neural basis of language can best be understood by the concept of *neural multifunctionality*. In this paper the term “neural multifunctionality” refers to incorporation of nonlinguistic functions into language models of the intact brain, reflecting a multifunctional perspective whereby a constant and dynamic interaction exists among neural networks subserving cognitive, affective, and praxic functions with neural networks specialized for lexical retrieval, sentence comprehension, and discourse processing, giving rise to language as we know it. By way of example, we consider effects of executive system functions on aspects of semantic processing among persons with and without aphasia, as well as the interaction of executive and language functions among older adults. We conclude by indicating how this multifunctional view of brain-language relations extends to the realm of language recovery from aphasia, where evidence of the influence of nonlinguistic factors on the reshaping of neural circuitry for aphasia rehabilitation is clearly emerging.

## 1. Introduction

The purpose of this review paper is to provide an update and summary of research from lesion studies, neuroimaging, and developmental studies of language in the aging brain focusing on converging evidence regarding the highly interactive relationship between linguistic functions and other cognitive functions. A clear and comprehensive model explaining the functional neuroanatomy of language in the neurologically intact brasin is still a work in progress. The newest attempts to propose such models represent a consistent shift towards accounts with increasing empirical and conceptual resolution that aim to capture the* dynamic* nature of the biological foundations of language (e.g., [1–4]). Better empirical resolution is now being accomplished through the enhanced level of detail with which temporal and spatial features of language-related brain activation patterns can be examined. Greater conceptual resolution involves the increasing level of specificity with which representations/operations underlying different language functions can be described.

In this review, we argue that sufficient evidence exists to support the following hypothesis: a comprehensive contemporary model of brain-language relations can best be based on the concept of neural multifunctionality, that is, neural networks specialized for cognitive, affective, and praxic activity constantly and dynamically interact with neural networks specialized for language to support and ultimately create language as we know it. We introduce emerging multifunctional approaches to the neurobiology of language that call for the incorporation of nonlinguistic cognitive functions into language models of the intact brain as a theoretical foundation for understanding aspects of neural changes in aging and neural mechanisms of recovery from aphasia.

This paper is organized as follows: (1) a brief review of current models of the functional neuroanatomy of language, with reference to lesion and neuroimaging findings; (2) a summary of evidence from functional neuroimaging studies of persons with and without aphasia and studies of the aging population exploring the effects of executive system functions on aspects of language processing; (3) a discussion of practical clinical consequences of the concept of neural multifunctionality in recovery from aphasia; (4) concluding remarks in which we outline possible ways in which a neural multifunctional language system might work.

## 2. Models of Functional Neuroanatomy of Language: From Lesion Studies to Neuroimaging

Current brain-language models emerged in response to the classical Broca-Wernicke-Lichtheim-Geschwind lesion-deficit model of aphasia [5]. In this model, language areas were localized in left-lateralized manner, with certain regions being predicted to lead to specific patterns of language impairment following brain damage. Thus, for example, the left posterior inferior frontal region, Broca's area, was linked to speech production (where brain damage would result in articulatory problems); the left posterior temporal region, Wernicke's area, to auditory speech recognition (where damage would yield impaired language comprehension); and the* arcuate fasciculus* connecting these anterior and posterior regions to repetition (where damage would impair production by repetition but preserve comprehension).

This schematic view of brain-language mappings has given rise to clinical classifications of aphasic syndromes, which to this day continue to guide aphasia research and clinical practice in many circles. Seven major aphasic syndromes have been proposed, with varying behavioral patterns and lesion loci (e.g., [6, 7]). Over time, however, serious clinical, biological, and psycholinguistic inadequacies of these mappings were identified (e.g., [8–12]). These include, for example, failure to account for the wide range of lesion-deficit patterns observed in aphasia (e.g., when a lesion to a certain area does not necessarily result in a predictable behavioral profile, or when lesions to multiple regions result in behavioral patterns that would otherwise be predicted for a different area altogether) or an inability to explain changes in behavioral patterns observed in aphasia over time (e.g., when a person first diagnosed with Wernicke's aphasia presents later, in the chronic stage, with conduction-like behavioral patterns and/or anomic-like patterns). These changes are reportedly experienced by 30%–60% of patients [13], with anomia being the most common end result of all aphasia-producing lesions [13, 14].

Limitations of the classical model have been highlighted even further with the explosion of new findings emerging from studies using advanced techniques for measuring real-time brain activity, for example, hemodynamic changes in the brain through functional magnetic resonance imaging (fMRI), intrinsic brain connectivity through resting-state fMRI, or the time course of brain activation during task performance via electroencephalography (EEG) or magnetoencephalography (MEG). With these techniques, many new inter- and intrahemispheric language-related neural networks have been identified (e.g., [15–18]), extending well beyond the core language areas (e.g., [19–23]), including cortical networks bilaterally (e.g., [12]), as well as subcortical circuits [24–27].

Price [28], for example, in a review of standard coordinates of peak activations found in over 100 fMRI studies published in 2009, identified an intricate web of neural networks, mediating different processes implicated in language comprehension and production. These included the following brain-language mappings: activation of the superior temporal gyri bilaterally for prelexical acoustic analysis and phonemic categorization of auditory stimulus, middle and inferior temporal cortex for meaningful speech, left angular gyrus and* pars orbitalis* in for semantic retrieval, superior temporal sulci bilaterally for sentence comprehension, and inferior frontal areas, posterior* planum temporale*, and ventral supramarginal gyrus for incomprehensible sentences (e.g., as a measure of plausibility). Speech production was found to activate additional neural networks, including left middle frontal cortex for word retrieval, independently of articulation; left anterior insula for articulatory planning, left putamen, presupplementary motor area, supplementary motor area, and motor cortex for overt speech initiation and execution; and anterior* cingulate* and bilateral head of* caudate* nuclei for response suppression during monitoring of speech output. Such data have clearly stimulated a need to create new models of the neuroanatomy of language, with greater neural and psycholinguistic specificity. Ideally, such models would spell out the specific links between formal operations associated with certain language functions, as well as the dynamic spatial and temporal neuronal pathways mediating them [29].

### 2.1. Current Models: Where Neurology and Psycholinguistics Meet

Over the past 20–25 years attempts have been made to reconcile neurological data with psycholinguistic research in order to formulate a systematic account for the biological underpinnings of language (e.g., [4, 11, 19, 21, 22, 28, 30–35]). These new models have largely identified different functional anatomies related to particular word- and/or sentence-level linguistic processes with varying degrees of neural and/or psycholinguistic specificity.

#### 2.1.1. The Dorsal/Ventral Model (Hickok and Poeppel, [12, 36])

**Figure 1:**
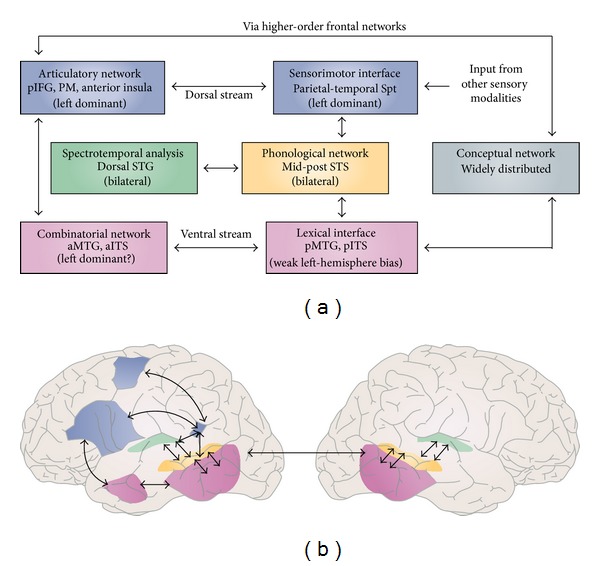
Dorsal-Ventral Streams (Adapted from Hickok and Poeppel [12]). STS: superior temporal sulcus; STG: superior temporal gyrus; aITS: anterior inferior temporal sulcus; aMTG: anterior middle temporal gyrus; pIFG: posterior inferior frontal gyrus; PM: premotor cortex.

One of the most influential proposals, already incorporated into current aphasia recovery studies (e.g., [37, 38]), is the dorsal/ventral model put forth by Hickok and Poeppel [12, 36]. This model uses a dual-route neuroanatomical architecture—dorsal and ventral streams—borrowed from the field of visual processing [39, 40] and from animal models of auditory processing in primates [41] to explain how auditory language proceeds. The ventral stream, also known as the “what” stream, is implicated in auditory recognition processes required for language comprehension, such as lexical semantic processing, mediated by neural networks projecting to different regions in the temporal lobe. The dorsal stream, termed the “where” stream, provides an interface for auditory and motor processing by performing phonological mappings of sound-to-articulatory representations, subserved by projections from auditory cortical circuits to temporoparietal and frontal networks. This architecture is shown in Figure 1.

Although the dorsal/ventral model offers a systematic neural account of the integration of auditory and motor information, it leaves open the computational nature of frontal networks, which have been assumed to interact with the dorsal system [33].

#### 2.1.2. The Psycholinguistics of Frontal Networks

The characterization of the functional neuroanatomy of frontal language networks has been the target of many psycholinguistic studies (e.g., [42]), which have offered different and sometimes opposing views of the processes implicated and their neural correlates (e.g., [19, 43]). These accounts largely differ in the extent to which they consider language to be a computationally independent component of the brain, that is, modular [44]. That is, they disagree about “whether there are domain-specific modules associated with different components of the grammar, whether such modules recruit distinct neural structures that are solely dedicated to the processing of that module and whether the neural systems associated with language are different from those recruited across other cognitive domains” [1, page 45].

Detailed proposals have been offered, linking particular frontal networks to specific aspects of semantic and syntactic processing (e.g., [32, 45, 46]), pointing to* fixed* module-specific neural architectures [1]. Friederici [24], for example, demonstrated a subdivision, according to which neural networks activated in “Broca's area,” specifically* pars opercularis* and* pars triangularis* (areas BA 44/45), support the reconstruction of sequential input into hierarchical syntactic structures during language comprehension, while BA6 and the frontal* operculum* support the processing of local structures. Her analyses consider brain activation in response to sentence comprehension tasks involving canonical and noncanonical word orders of varying lengths and processing demands, as well as syntactic violations at the phrase level.

In contrast, Hagoort [43] has argued for a model that implies the operation of* distributed* neural networks, in which language processing (comprehension and production) in Broca's area, the left inferior frontal gyrus (LIFG), involves parallel processing of semantic, syntactic, and phonological information, accomplished via three functional components: memory, unification, and control, memory, to retrieve language information stored in long-term memory, unification, to integrate the retrieved information into larger (multiword) units, and control, to select what he terms a language “action.” Using evidence from EEG and MEG studies, he has been able to identify the specific temporal features of unification and memory retrieval, arguing for neuronal synchronization that supports functional interrelatedness rather than strict domain specificity [47].

#### 2.1.3. The Need to Consider Nonlinguistic Functions

We are not proposing here to adopt Hagoort's framework for the study of the neural organization of language but rather to suggest considering the importance of at least one implication of his model that, at any given time, the processing of linguistic information is necessarily affected by the processing of other types of information. This implication rules out a strict modular view of language, where discrete neurofunctional components rather than multiple functionally overlapping neural networks are postulated [1].

Indeed, functionally diverse neural networks have been identified in the LIFG (e.g., [33]), including language functions, such as speech processing (e.g., [48]), processing of syntactic complexity [19], semantic processing [49], and plausibility (e.g., [50, 51]). Moreover, and importantly, these frontal networks have also been linked to a number of nonlinguistic functions, including, but not limited to, processing of math operations, mental rotations, and music [52–55]. Comparable claims have been made regarding left temporal networks, whose posterior portions, for example, have been found to be involved in syntactic processing (e.g., [56–58]) as well as in nonsyntactic tasks (e.g., [58–60]).

Researchers have been able to isolate some of the frontal networks subserving these apparently overlapping functions, supporting perhaps a weaker version of modularity, that is, a* multifunctional modularity* [61] approach to language, in which independent functional components and their neural correlates can be identified and then incorporated into a model that would tie them together. Such a view would be able to account, for example, for findings such as those of Makuuchi and colleagues [62], who described an anterior-to-posterior functional architecture within the prefrontal cortex, supporting a domain-general hierarchical structure, shared across language, arithmetic, and working memory tasks, but with the dorsal* pars opercularis* being specifically dedicated to the processing of hierarchically complex sentences (evidenced by patterns of reduced brain activation in the* pars opercularis* in response to the language tasks).

### 2.2. Multifunctional Brain-Language Models: Work in Progress

The findings described above call for an integrative brain-language model that accounts for multifunctionality across shared neural networks [1]. A multifunctional neural model of language would require the mapping of brain-language architectures that captures the functional diversity of the neural networks mediating language, including the functional contributions of nonlinguistic skills. Fedorenko et al. [63] state it nicely: “In order to claim that a particular brain region R supports a particular cognitive function, it is necessary not only to formulate predictions about the kinds of cognitive operations that should result in activity in region R, but also to be able to explain why other kinds of cognitive operations result in activity in region R” (page 188). As we will show, this is a task easier said than done. The enormity of the challenge lies, in part, in the difficulties defining the nature of these nonlinguistic contributions and their own neural bases. Carpenter et al. [64], for example, have pointed out that the cortical organization of executive functions and working memory are widely and dynamically distributed in regions extending beyond prefrontal areas, making it difficult to clearly identify the specific mechanisms allocating functions to particular neural regions.

Nonetheless, in the most recent models of the functional neuroanatomy of language (e.g., [21, 22, 34, 35, 65]), efforts to identify several neural interfaces among language, cognitive, motor, and sensory processes have been made. Friederici [21], for example, has proposed a model comprising at least two dorsal and ventral streams [35], which support the processing of spoken language, from auditory perception to sentence comprehension and interact at certain points with working memory in the process. Her arguments are largely based on neuroimaging and electrophysiological studies, where carefully designed language tasks with specific contrasting features (e.g., comparison of words and pseudowords, or semantically plausible sentences to implausible ones) have been used to create highly specified brain maps for phonological, semantic, and sentential processes.

The two ventral pathways are assumed to mediate semantic information processing (e.g., word-level semantic categorization, lexical-semantic access, and sentential plausibility), via networks implicating BA45/47 and the frontal* operculum*, as well as basic syntactic operations (e.g., local phrase structure building), through the* uncinate fasciculus* (UF) connecting the frontal* operculum* and temporal regions. The empirical validity of this proposal needs to be qualified, however, by the observation that the UF has been linked to language functions (naming) only in a small number of studies [66, 67]. Others have found that resection of the UF has limited effects on the long-term language functions, such as sentence processing (e.g., [68]) and semantic processing (e.g., [69]).

The two dorsal tracts in this system are assumed to subserve sensory-to-motor mappings involving the temporal cortex, the primary motor region, and the* pars opercularis* (area BA44), as well as the processing of structurally complex sentences, where information is transferred from BA44 to the posterior temporal cortex, in a top-down fashion (e.g., when examination of sentential context is called for). Because activation of both BA44 and the temporal cortex has been observed during syntactic processing and because the dorsal portion of BA44 and the inferior frontal sulcus have been linked to syntactic working memory, Friederici has suggested incorporating working memory into her model as a functional support to the processing of syntactically complex sentences (in line with the works of [70, 71]). However, the exact cognitive mechanisms linking prefrontal and parietal regions, which also need to be integrated to allow sentence comprehension to occur, are left tentative. Using dynamic causal modeling, Makuuchi and Friederici [72] have tried to clarify this issue by analyzing the processing of complex syntax during reading. They proposed hierarchical connectivity according to which processing of linguistic information proceeds from visual word form regions (fusiform gyrus) through working memory areas (inferior frontal sulcus and intraparietal sulcus) to language regions (*pars opercularis* and the middle temporal gyrus), with greater connectivity found as processing load increases.

A more comprehensive model of the functional neuroanatomy of language, which considers nonlinguistic components, has been put forth by Price [22], who reviewed over 1000 positron emission and neuroimaging studies published over the past 20 years (1992–2011). She found converging evidence for neural networks supporting heard speech, speech production, and reading, which are affected both by sensory and motor processes localized to specific structures and by distributed activations shared across several functions.

**Figure 2:**
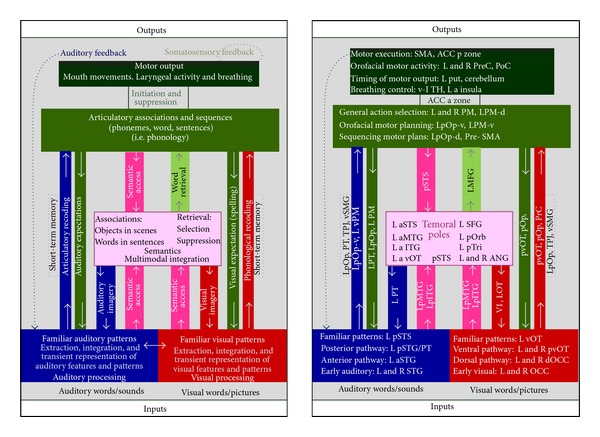
Brain-Function Mappings (Adapted from Price [22]), a: anterior; A: auditory cortex; ACC: anterior cingulate; AG: angular gyrus; c:* caudate*; CB: cerebellum; d: dorsal; GP: globus pallidus; IFS: inferior frontal sulcus; IOG: inferior occipital gyrus; ITG: inferior temporal gyrus; MFG: middle frontal gyrus; MTG: middle temporal gyrus; Occ: occipital; OT: occipitotemporal; p: posterior; PO: parietal operculum; pOp:* pars opercularis*; pOrb: pars orbitallis; pTri: pars triangularis; PT: planum temporale; poC: postcentral; preC: precentral; PM: premotor; PUT: putamen; SFG: superior frontal gyrus; SMA: supplementary motor cortex; STG: superior temporal gyrus; STS: superior temporal sulcus; SMG: supramarginal gyrus; TPJ: temporoparietal junction; Th:* thalamus*; v: ventral; VI: lobule VI (medial anterior); VII: lobule VII (lateral posterior).

Price [22] analyzed neural data for nine major language functions, including auditory speech processing of sounds (speech and nonspeech); phonological processing; speech comprehension (semantic and syntactic); word retrieval; covert articulatory planning, overt articulation; auditory and motor feedback in speech production; visual word processing; and orthography-phonology mapping, parcellated into 36 cytoarchitectonic regions (see Figure 2). Each of these functions is proposed to interact with specific nonlinguistic processes, such as acoustic processing of all types of auditory stimuli; rate of transitions in rapidly changing auditory stimuli; short-term memory to maintain auditory imagery when no auditory input is available; influence of general multimodal context on sentence comprehension (e.g., to guide guessing); selection of motor commands from several options; ordering complex motor commands; and timing of motor output to ensure execution of motor plan, implicating particular neural networks.

The systematic brain-function mappings Price has identified are described in Figure 2 (adapted from [22], Figures  2 and  3).

These complex interdependencies speak to the integrative nature of Price's model, where multiple networks are recruited in service of language processing (e.g., phonologic or orthographic) and supported by the functional integration of multiple bottom-up and top-down processes.

However, as Price [28] herself has pointed out, there may be more to the circuitry of language networks in the brain than meets the “neuroimaging” eye. Most studies continue to explore specific functions associated with the usual cortical-cortical “suspects,” in spite of a growing appreciation of the involvement of additional neural structures in the mediation of different language processes. Little is still known about language networks in the* cerebellum*, which have been found to be activated in response to articulation (e.g., [73]), acquisition of novel words (e.g., [74]), auditory self-monitoring (e.g., [75]), and working memory (e.g., [76]), or networks associated with subcortical circuitry, whose support of language functions has often been reported to be bilateral (e.g., [28]). There is evidence implicating, for example, the left* caudate* in the control/selection of motor sequences necessary for articulation, which has been argued to be activated even for language comprehension tasks, when less automatic processing of input it called for [24]. The “control” function of the* caudate* has been associated with a neural circuit linking the* caudate* to prefrontal, premotor, and temporal and parietal cortices reciprocally through the* thalamus*.

#### 2.2.1. Where Do the Current Models Fall Short?

One of the arguments raised against neural models of language such as those just discussed is that they overlook the considerable neuroanatomical differences observed in the intact brain, which limit the ability to reliably describe a clear functional neuroanatomy for language (e.g., [63, 77]). The rationale underlying this claim is that most neuroimaging studies of language rely on neural data derived from meta-analyses or group analysis maps, which fail to capture the full scope of brain activity in specific target regions (e.g., when brain activation patterns in a voxel-based analysis are averaged over participants in whom no effects are found), underestimating these regions' functional specificity [78]. Instead, Fedorenko et al. [77] have developed a “functional localizer,” modeled after neuroimaging techniques used in other domains, including vision [79] and social cognition [80]. This method allows for a quick mapping of language-sensitive regions* within* an individual which could then be pooled* across* individuals to delineate* functional*, rather than anatomical, regions of interest, and so circumvents the problem of interindividual variability.

However, as Grodzinsky [61] has pointed out, the source of interindividual variability that Fedorenko and colleagues highlight is not strictly neuroanatomical but derives, in part, from the choice of linguistic tasks selected to demonstrate functional distinctions. He also adds that in spite of such individual differences, group results obtained in neuroimaging studies paint a robust and clear picture (evidenced, e.g., in Price's [22] work), where “we accomplish localization at the best currently available resolution” ([61, page 614]).

Is the resolution of current neuroimaging techniques sufficient for unveiling the unknowns of the functional neuroanatomy of language? It is possible that our limited ability to tease apart the intricate cortical-subcortical underpinnings of language functions has to do with the fact that most of the analyses conducted are focused on cytoarchitectonic parcellation of brain structures* alone*. Such analyses miss the potential contributions of (1) neurochemical mechanisms of neurotransmission to language functions and (2) factors of psycholinguistic task selection. Our concern here finds support in the work of Amunts et al. [33], who have uncovered a novel organizational architecture of the frontal cortex at the neuronal level, based on a multireceptor analysis of brain tissue. This circuitry included connections among premotor, prefrontal, and Broca's cortices, involving previously unexplored neural structures, with a strong left lateralized of cholinergic receptors (M_2_) in the dorsal and ventral areas 44v and 44d.

Examination of language at the neuronal level has already started gaining popularity both in studies of the healthy brain (e.g., [47]) and in studies of treatment-based changes in language performance during aphasia recovery (e.g., [81]). The premise of these studies is that abstract language models are inherently unable to detail neuronal circuitry and therefore have little utility for neurobiological studies of language (e.g., [81]). Instead, such investigations have relied on models that directly simulate brain activation, for example, models of parallel distributed processing (PDP), which, by and large, assume that the most basic functional unit is “the neural network,” which consists of operational units that correspond to firing rates of neurons, whose spreading activation gives rise to a given behavior (e.g., [82]). This neural activity is assumed to reflect experience-based statistical regularities that account for a range of cognitive functions rather than independent operations of grammatical composition.

However, to explain the neurobiological foundations of language, there is no need to appeal to the irreconcilable gap between abstract linguistic notions and neural data [83]. The lens should be directed at the distinct temporal and spatial features underlying functional relations [84, 85], where neuronal groupings cluster in combinations within and outside of cortical networks, to yield specific operations/computations [3].

## 3. Interactions between Executive System Functions and Language in the Brain

Because damage to lateral portions of the left prefrontal cortex has also been found to lead to language-related* executive control* deficits, including impaired verbal fluency [86], poor monitoring of verbal information over short periods [87], poor concept shifting [88], and difficulties with complex planning [89], attempts have been made, especially over the past two decades to understand these neurofunctional interdependencies in the healthy brain, examining executive effects on specific language functions, such as sentence processing and lexical retrieval. By way of example, we consider here the effects of executive system functions on aspects of semantic processing.

### 3.1. Semantic Control in the Intact Brain

Psycholinguistic research has placed a particular focus on characterizing the neural representation of* semantic control*, which activates (as opposed to stores) semantic knowledge through cognitive control processes (e.g., [90–98]). Specifically, this phenomenon refers to a two-step process by which a given word meaning is retrieved and then selected among several semantically related target competitors. Controlled retrieval happens as we search for information that may be of relevance,even if only remotely related to the target, when the semantic information in the stimulus is insufficient to help identify the target or when task-relevant information is not activated. Controlled selection follows controlled retrieval and aids in the selection of the item with the most goal-appropriate characteristics in the face of several activated target-related competitors available for selection (e.g., [96, 98, 99]).

The functional neuroanatomy of semantic control has been associated with neural networks in the left inferior frontal gyrus (LIFG) [100–102]. These networks allow for retrieval and selection of semantic and other types of knowledge (e.g., [98, 103, 104]), evidenced, for example, by reduced brain activation when automated semantic associations are performed [105] or increased activation when distant semantic relations among stimuli are processed [95, 96], even when response times are matched [106]. Support for this claim can be found in a recent study in which a virtual lesion was induced in the LIFG through transcranial magnetic stimulation leading to both retrieval (identification of weakly associated words) and selection (detecting features in the presence of strong distractors) problems [107].

A distinction can be drawn between anterior ventral portions of the left inferior prefrontal regions and its posterior dorsal region, which are assumed to subserve controlled used of semantic and phonologic information, respectively (e.g., [94, 108, 109]). The precise nature of the control processes associated with these regions is currently debated, with some arguing that the left inferior frontal gyrus (LIFG) mediates selection rather than retrieval [110], while others claim that both selection and retrieval are supported by the region [104]. Some findings have indicated that recruitment of temporoparietal networks is also necessary for semantic control [107, 111] but that their role is distinct from those of the LIFG networks [99]. It has been proposed, for example, that the LIFG suppresses previously presented relevant semantic information, whereas the temporoparietal networks, in concert with LIFG, help retrieve less dominant semantic information to match task-relevant information [112].

Ventral white matter tracts connecting frontotemporal regions, especially the projections of* uncinate fasciculus* (UF) and the inferior longitudinal* fasciculus* (IFG), have also been reported to be involved in semantic control processes, evidenced by performance on a homonym meaning decision-making task [113]. IFG projections, however, have also been found to be involved in processing meaningful speech (e.g., [114, 115]) and so may not be uniquely specialized to mediate semantic control processes.

Other studies have demonstrated involvement of cortical-subcortical circuitry in mediating executive-language function dependencies (e.g., [27, 116–118]). For example, four corticothalamic and thalamic-cortical mechanisms have been identified as crucial “executive” supports for language functions, at least at the word level: (1) frontal cortex's selective engagement of cortical areas in an “attentive” state relevant to task performance via the* nucleus reticularis*, (2) transfer of information from one cortical area to another through corticothalamocortical relays, shifting attention as necessary, (3) optimizing focus on task-relevant information through corticothalamocortical mechanisms of feedback to ensure, for instance, processing accuracy, and (4) word selection during the expression of a concept whereby signal-to-noise ratio increases around the selected word, mediated by a* basal ganglia* loop [27].

These mechanisms are assumed to support intentional functions, with intention referring to the ability to select and initiate an action among several competing options (as opposed to attention involving the selection of a stimulus among competing stimuli and further processing that stimulus) [27]. Specifically, the neural representation of intention is thought to involve the supplementary motor area (SMA), pre-SMA, rostral* cingulate* area, lateral frontal regions, and* basal ganglia* loops [119], with pre-SMA, dorsal* caudate* nucleus, and ventral anterior* thalamus* mediating generation of meaningful but not nonsense words [120], or word repetition [121]. Within this architecture, the pre-SMA is assumed to generate an automated word selection bias which is then maintained by the* basal ganglia*, affecting top-down processing during word selection [122].

### 3.2. Semantic Control in Aphasia

Studies describing difficulties in semantic control among people with aphasia also provide crucial evidence for the contribution of an executive component with its specialized neural correlates to semantic processing (e.g., [97, 123, 124]). For example, researchers have compared the performance on semantic processing tasks with varying task demands between people with semantic dementia (SD) and those with stroke-based aphasia (e.g., [97, 123, 124]). Both patient groups were found to perform poorly on tasks that require processing of semantic memory in tasks involving semantically related competitors but differ in their ability to control variable task demands. People with SD showed good control, resulting in item consistency across different task demands, as opposed to persons with aphasia, who performed consistently only when task demands were kept constant (e.g., [97]). Disruption in the ability of persons with aphasia to manipulate semantic knowledge flexibly in the face of changing task demands was found to be eliminated when phonemic cueing was provided [123], highlighting the dissociation between impaired control abilities and preserved stored semantic knowledge. The sensitivity of persons with aphasia to executive task demands has also been demonstrated in nonverbal domains, including difficulties in nonroutine usages of everyday objects and improved performance under more structured task conditions accompanied by verbal and visual cues (e.g., [125, 126]).

Impaired semantic control has been observed among persons with aphasia with damage to left prefrontal cortical circuits. Thompson-Schill and colleagues [127], for example, reported that patients with left inferior prefrontal lesions implicating neural substrates in Brodmann's BA 44, but not those with prefrontal lesions excluding these neural substrates or patients with right hemisphere damage, show very poor performance on noun selection tasks with high competing demands, arguing for a selection among competitors deficit. Aphasic patients with damage to temporoparietal networks have also been shown to have difficulties with semantic control (e.g., [97, 128, 129]), although greater impairments have been observed in patients with anterior lesions [112, 130].

The deficits observed among persons with aphasia with prefrontal lesions have been shown to affect their performance on tasks involving cumulative competition across cycles, as stimuli items constitute both targets and distractors on different trials [112]. The ability to navigate through such tasks largely depends on whether the control network can generate timely task-appropriate responses that activate semantic information within the semantic store, which becomes increasingly difficult in the face of strong competition and/or open-ended task demands (e.g., [99, 110, 111]), leading to reduction in accuracy in both verbal and nonverbal modalities [112]. It has been proposed, then, that the neural substrates of the left inferior frontal gyrus (LIFG) specifically mediate selection among items that have already been retrieved (e.g., [131]), affecting even sentence production tasks in which the probe refers to several propositions [132].

Using diffusion tensor imaging (DTI) and resting-state functional magnetic resonance imaging (rs-fMRI) data obtained from persons with aphasia in a study of semantic control abilities, Harvey et al. [133] found that the white matter tracts connecting frontotemporoparietal regions were directly related to impaired word comprehension involving control processes, where structural integrity and strength of functional connectivity of* uncinate fasciculus* (UF) predicted semantic control abilities among the participants. Specifically, patients in whom decreased structural integrity and weaker connectivity of UF but no significant damage to anterior temporal and inferior frontal pathways themselves were observed also performed poorly on word comprehension tasks (the ability to correctly reject semantic foils and the ability to retrieve semantic knowledge about an item while ignoring other semantic relationships).

Subcortical circuitry has also been described as an intersection of language and executive impairment in aphasia, although most studies of thalamic aphasia do not provide behavioral data regarding performance on tests of executive functions. An exception is a study by Radanovic et al. [117], who found that left, but not right, thalamic lesions implicating corticothalamic-cortical reciprocal connections can result in failed semantic control, where the ability to differentiate semantically related words is disrupted by poor executive control, as measured, for example, by low scores on executive function tasks such as Trail Making and the Wisconsin Card Sorting Task, leading to anomia or paraphasic misselections. The authors attributed this finding to a formulation deficit adversely affecting language organization and conceptual association. Among their participants with right thalamic lesions they found different problems affecting visuospatial perception with concomitant problems on discourse script tasks, especially temporal-sequential ordering [117], reflecting more a “thought” disorder, independent of language impairment [134]. Other studies of behavioral profiles among aphasic people with thalamic lesions (e.g., [135]) have relied on sophisticated test batteries, designed to differentiate levels of word processing deficits—lexical, semantic, lexicosemantic—to identify the precise level at which deficits are demonstrated (lexicosemantic). The reader is referred to an excellent review by Crosson [27], in which the behavioral manifestations and neural mechanisms implicated in thalamic aphasias are discussed in detail.

In sum, results from neuroimaging studies and studies on aphasia, despite their methodological flaws and inherent limitations, converge on the following notion: semantic processing and its neural bases do not exist in isolation from constant and dynamic interaction with executive system function and its neural bases.

### 3.3. Interactions of Executive System Functions and Language in the Aging Brain

Modeling of the functional neuroanatomy of language has clearly come a long way since the days of the classical Broca-Wernicke-Lichtheim-Geschwind model, both in terms of its neural resolution as well as in its empirical scope. However, all too often the proposed brain-language maps are based in large part on neuroimaging data collected from young healthy adults (e.g., usually college-aged students), whose functional neuroanatomy is unlikely to map onto that of older adults in a one-to-one fashion. As we briefly discuss below, there is evidence suggesting that language and some of its related nonlinguistic supports do not remain constant throughout the lifespan.

Progressive decline in age-related language functions typically involves difficulties with lexical retrieval and sentence processing, even if sometimes subtle. Older adults' reduced ability to retrieve nouns and verbs, for example, has been linked to problems in accessing phonological forms of words (e.g., [136–145]). And their decreased sentence processing abilities (lower accuracy and/or slower reaction times) have been argued to be affected by syntactic complexity, low plausibility, decreased predictability, or increased background noise (e.g., [146–155]).

Efforts to explain these linguistic declines have mostly appealed to neurocognitive changes observed with age, such as overall reduction in processing speed (e.g., [156–158]) or degradation of specific cognitive functions, such as working memory, divided attention, inhibitory control, or set shifting (e.g., [153, 159–163]). Thus, older adults' slower processing speed has been argued to negatively affect their picture-naming abilities (e.g., [164]), especially when they are asked to name actions, as contrasted with objects [165, 166]. Or, their reduced working memory span, which arguably diminishes their ability to simultaneously store and process information (e.g., [159]), has been argued to impair their accuracy on syntactically complex sentences (e.g., processing stimuli containing embedded clauses or more than a single negative marker) (e.g., [153]). By the same token, successful language performance among older adults has been linked to the sparing of cognitive abilities, where the combined contribution of preserved cognitive functions reflects a compensatory mechanism recruited to support a given compromised linguistic function (e.g., [167, 168]), with the better-performing adults being those in whom a greater number of cognitive functions are preserved (e.g., [157, 169]).

These age-related compensatory mechanisms have been correlated with particular neural changes in hemispheric asymmetry observed with age (e.g., [169, 170]), resulting from changes in gray matter volume and/or white matter integrity (e.g., [167, 171–175]). A current consensus among researchers working in the field of language in aging is that language functions among older adults increasingly rely on support networks outside traditional core language networks, extending to right homologous counterparts (e.g., [167]). In neuroimaging studies exploring the neural circuits associated with lexical retrieval among older adults, for example, frontal bilateral involvement has been linked to action and object naming tasks and certain list generation tasks [174–176], with some variability in the particular brain regions implicated, based on task type used in each study (e.g., [177]). Comparable claims have been made in studies considering patterns of brain activation in relation to sentence processing tasks (e.g., [21, 50, 167, 178–183]), where more widespread brain activation is consistently described.

Advances in technology allowing closer examination of brain activity in real-time, improvements in the experimental design applied to neuropsychological studies, and the development of psycholinguistically motivated theories of language have opened novel and exciting ways of exploring the functional neuroanatomy of language. Neuroimaging data from young and older adults clearly suggest that key neural networks dedicated to language functions partially subserve nonlinguistic functions, such as executive system function, working memory, or attention control, which contribute reciprocally to aspects of language performance, even if, at present, the extent of overlap between models based on young brain data and those describing the aging brain remains underspecified.

## 4. Neural Multifunctionality and Recovery from Aphasia

To this point in the paper we have been providing converging evidence from divergent strands of current research to support the notion that language as we know it cannot be dissociated from its constant and dynamic interaction with nonlinguistic functions and that the neural basis of language is intimately linked, at all times, with neural networks supporting cognitive and emotional functions, within a theoretical framework we are calling neural multifunctionality. For this paper, we have been exploring and reviewing interactions—both cognitive and neural—between executive system function and language. Abundant evidence exists to demonstrate the same constant and dynamic interaction of language with attention, memory, praxis, visuospatial function, and affective behaviors. If our thesis is correct, it should be possible to develop therapy programs for persons with aphasia that focus on rehabilitation of nonlinguistic functions that are considered to be intimately linked to specific language functions. Such programs are, in fact, being developed.

One such program explores how targeting language support systems, in particular executive functions, can affect brain reorganization in chronic aphasia. This technique studies the effects of incorporating “intention treatment,” which targets neural mechanisms responsible for action initiation, into the treatment of naming deficits [184–186].

In an fMRI study of two people with residual nonfluent aphasia who received an intention treatment and a comparable attention treatment without an intention component, Crosson et al. [184] demonstrated treatment-based neural reorganization of language functions in posterior persylvian regions. Because intention refers to the ability to select and initiate and because nonfluent aphasia can involve problems with word selection and initiation of output, the authors proposed characterizing aphasia as a disorder of intention, predicting benefits in response to the intention but not attention treatment. In support of their choice, they cited behavioral studies reporting picture-naming gains following intention treatment, compared to baseline performance [187].

Crosson et al. [184] treatment protocol involved the initiation of a word finding trial with a left-hand motion on the left side (lifting a lid to press a button in a box, or repeating the target stimulus after the examiner, using a nonsymbolic circular left-hand gesture, if performance was incorrect). This initiation sequence was designed to activate right medial frontal intention mechanisms, on the assumption that poststroke right frontal brain activation reflects attempts of the right hemisphere to perform language functions. The authors further assumed that continued activation of left-medial structures—the presupplementary motor cortex—can suppress this right hemisphere activation, via the right* basal ganglia*, leading to inefficient processing of linguistic information required for word production. Their objective in this treatment was therefore to reduce this inefficiency by shifting the activity to the right pre-SMA and the right lateral frontal region.

Their patients, however, showed differential responses to these treatments, with one benefiting from both treatments, compared to the other, who responded only to intention but not to attention treatment. To explain this finding, the authors appealed to neuroanatomical differences between the two patients, resulting in distinct mechanisms of neural plasticity. The patient who responded to both treatments had a lesion that spared the left* basal ganglia* and* thalamus*, allowing for a natural pretreatment right hemisphere reorganization of language functions, where the left* basal ganglia* continued to suppress the tendency to activate the left frontal mechanisms. In the patient who responded only to intention treatment these subcortical structures were damaged, blocking the natural transfer of word production abilities, enabled by intention intervention (triggered by left-hand movements). Because of this subcortical damage, continued left hemisphere activation could not be suppressed, requiring even greater activation of right hemisphere frontal mechanisms for this inhibition. Crosson et al. [184] thus proposed that extent of* basal ganglia* lesion could help determine the need to include an intention component in therapy to promote functional recovery of language in aphasia.

In sum, whether neurorehabilitation approaches for aphasia involve manipulation of executive system functions or some other aspects of nonlinguistic manipulation, clearly the field is wide open for new approaches to therapy based on the principle of neural multifunctionality.

## 5. Conclusions

Taken by themselves, each of the methodological approaches we have reviewed in this paper is flawed, each for its own reasons. Taken together, results from neuroimaging, lesion studies, and studies of language in the aging brain provide compelling converging evidence for the concept of neural multifunctionality, a concept that has both theoretical and practical/clinical implications—theoretical with regard to models of brain-language relations and practical with regard to rehabilitation of persons with cognitive deficits as a consequence of brain damage.

The question remains, of course, of how a neurally multifunctional language system might work. Borrowing from recent developments in the memory literature [188], which emerged, in part, to account for apparent overlaps between the neural substrates mediating “what” and “how” memory functions (e.g., [98, 189, 190]), we propose to adopt a* component process framework *to language processing.

Under such a framework, linguistic information would be processed through a neural system of* component processes*, in which region-specific neural configurations contribute to multiple cognitive tasks simultaneously. The component interactions are conceived as “process-specific alliances.” These alliances are small brain regions temporarily recruited to accomplish a cognitive task, given specific task demands. Each component in the alliance has a specific function, and they combine together to give rise to a complex operation. These small neural “groups” disintegrate once task demands are met and are thus distinct from larger-scale networks, whose connectivity continues to be observed at rest [191–193]. The links among the components in the stable larger-scale networks can affect which alliances are formed, but they do not directly determine them. This approach is aligned with our view of neural multifunctionality of language, whose operations rest on the interaction of “neural cohorts” subserving multiple functions in cognitive, emotional, motor, and perceptual domains.

The neural multifunctionality approach we propose here will allow the reevaluation of current concepts of recovery from aphasia, focusing on the* dynamic* development of new neural support systems in the aphasic brain in service of new functions. We propose that this multifunctionality operates in a multidirectional and reciprocal fashion, such that neural networks engaged in language recovery mutually interact with neural supports of nonlinguistic functions so as to give rise to* new* functional neuroanatomies (i.e., newly established or newly reinforced neural networks) in the neurologically compromised brain.
